# Dietary substitution of soybean oil with coconut oil in the absence of dietary antibiotics supports growth performance and immune function in nursery and grower pigs

**DOI:** 10.1186/s40104-020-0428-4

**Published:** 2020-03-16

**Authors:** Andrea G. Braundmeier-Fleming, Cassandra S. Skenandore, Lesly Gil, Victoria Jacobsen, Melissa Cregger, Taylor Badger, Mallory Karr, Guoyao Wu, Stephen B. Smith, Annie E. Newell-Fugate

**Affiliations:** 1 Department of Medical Microbiology, Immunology and Cell Biology, School of Medicine, Southern Illinois University, Springfield, IL 62702 USA; 2 Department of Veterinary Physiology and Pharmacology, College of Veterinary Medicine and Biomedical Sciences, Texas A&M University, 4466 TAMU, College Station, TX 77843 USA; 3 Department of Animal Science, Texas A&M University, 2471 TAMU, College Station, TX 77843 USA

**Keywords:** Alternatives, Antibiotic supplementation, Grower pigs, Medium chain fatty acids, Nursery pigs, Oil

## Abstract

**Background:**

We hypothesized that supplementation of nursery and grower pig diets with coconut oil in the absence of antibiotics would yield maintenance of glucose homeostasis, growth performance, and immune function similar to what is achieved with nursery and grower pig diets containing antibiotics. Pigs received the same base treatment diets from d24 (weaning) to d71 of age and had blood and fecal samples collected on d24, d31, d45 and d71 for measurement of whole blood glucose, serum insulin, cortisol and cytokines, and fecal microbiome. Pigs had weekly weights and daily feed consumption measured throughout the study. Animals were euthanized at d71 and subcutaneous fat and ileal contents were collected for assessment for fatty acids and microbiome, respectively. Diet treatments consisted of 2% soybean oil plus antibiotics (ABX; *n* = 22), 2% soybean oil without antibiotics (NABX; *n* = 22), and 2% coconut oil without antibiotics (COC; *n* = 22). Statistical analysis examined the effect of diet within each timepoint using a repeated measures ANOVA.

**Results:**

Pigs fed COC diet had decreased serum insulin levels, maintained feed intake, feed conversion and weight gain, and, based on serum cytokines and fecal microbiome, were immunologically similar to ABX-fed pigs. However, NABX-fed pigs performed similarly to the ABX-fed pigs in all parameters except for serum cytokines. Additionally, there was no difference in the incidence of diarrhea between any of the diet treatments.

**Conclusions:**

This study demonstrates that dietary antibiotics are not necessary to maintain growth performance in nursery and grower pigs. However, dietary antibiotics appear to modulate circulating cytokine levels. Dietary coconut oil is neither harmful nor helpful to growth performance or immune function in nursery and grower pigs but does modulate serum insulin levels. Therefore, while coconut oil fed at 2% by weight is a suitable substitute for dietary antibiotics, this study suggests that no substitute for dietary antibiotics is needed at all.

## Background

For decades, dietary antibiotic supplementation to control sub-clinical infections has been standard in the American swine industry thereby boosting pig growth, immune function, and overall health [1]. However, such practices have been implicated as one of the driving forces behind the development of antibiotic resistant bacteria [2]. In 2013, the FDA recommended that the pharmaceutical and livestock industries voluntarily phase out the use of antibiotics for growth promotion purposes by the end of 2016 [3]. In light of these facts, it has become more urgent to find a replacement for subclinical doses of antibiotics in both nursey swine pig diets, but as yet suitable replacements have not been identified. Coconut oil is comprised of 91% saturated fatty acids (FA), which are predominantly medium chain FA such as lauric acid. The exposure of immune cells to lipids with differing FA composition may alter immune cell function through multiple mechanisms including altered eicosanoid synthesis and inflammatory gene expression (i.e., NF-κB) [4]. In the cattle industry, dairy calves fed a commercially available milk replacer with coconut oil for a 56-day period had greater average daily weight gain and feed efficiency, reduced scours, and reduced medical treatment incidents for clostridium illness, compared to the control group [5]. Lambs supplemented with coconut oil from 15 days to 6 months of age showed improved feed conversion and similar carcass characteristics, compared to a non-coconut oil supplemented group [6]. Therefore, coconut oil holds promise as a viable method for modulation of immune function and growth which could maintain carcass quality similar to that found in animals fed a traditional diet. We hypothesized that coconut oil is an effective alternative method to antibiotic feed supplementation that would maintain or improve nursery and grower pig health and growth.

## Methods

### Animals, diets, and sample collection

All animal experimental procedures were approved by the Texas A&M University Institutional Animal Care and Use Committee (IACUC Number 2015–0385). Pigs were born and housed at the Texas A&M University Animal Sciences Teaching, Research, and Education Complex in College Station, Texas. At 24 d of age and an average body weight of six kg, Yorkshire-cross piglets (*n* = 66) were weaned, vaccinated with Parapleuro Shield™ P + BE (Elanco Animal Health, Greenfield, IN), had four mL of blood and ~ one mL of fresh feces collected, and were placed in indoor nursery pens of 5–6 piglets per diet treatment group with 4 pens (technical replicate) per diet treatment for a total of 22 piglets per diet treatment. The nursery was environmentally controlled for temperature (23–30 °C) and humidity (85–100%) and had a 12-h light:12-h dark cycle. Dietary treatment groups consisted of: 1) Antibiotics (ABX): soybean meal, cornmeal, 2% soybean oil, antibiotics; 2) No antibiotics (NABX): soybean meal, cornmeal, 2% soybean oil; 3) coconut oil (COC): soybean meal, cornmeal, 2% virgin coconut oil. The basal diet to which the oils and/or antibiotics were added consisted of three phases from nursery through grower development stages (Additional file 1: Tables S1, S2 and S3). Antibiotics added to the ABX diet were added as per product label dosages and instructions (i.e. growth promotion levels) for each antibiotic formulation used: Denagard® (tiamulin hydrogen fumarate; Elanco, Greenfield, IN, USA) at a rate of 0.175% of base diet (W/W) and Aureomycin® (chlortetracycline; Zoetis, Parsippany, NJ, USA) at a rate of 255 g per 907 kg of diet. A dietary analysis of fatty acid composition for each diet treatment was performed as described below (3–4 diet samples per diet per phase) (Additional file 1: Table S4).

Daily feed intake per pen on an as-fed basis was calculated as the difference in the weight of the feed (in kg) in each trough before and after feeding for each pen (feed consumption) each day. This weight was divided by the number of piglets per pen to get the average daily feed intake per piglet per day. Piglets were weighed weekly throughout the trial. Each of these feed intake and weight parameters was used to calculate average daily gain (ADG), average daily feed intake (ADFI), and the feed conversion ratio (FCR) [7].

### Sample collection

Piglets were monitored daily for outward clinical signs of illness or scours, neither of which was noted in any piglet from any treatment at any point during the study. Piglets had fasting blood and fecal samples collected on d24 (weaning), d31, d45, and d71 (euthanasia). On sample collection days, piglets were fasted 8 h and 6 mL of blood was collected from the cranial vena cava. Blood was immediately analyzed for whole blood glucose with the Precision Xtra glucometer (Abbott Laboratories, Bedford, MA) which has been validated for use in pigs [8]. Blood tubes were kept one ice and were centrifuged within 4 h of collection for retention of serum, and EDTA and heparin plasma, which were kept at − 20 °C until analysis. For each pig, ~ one mL of feces was collected directly from the rectum and placed in a 2-mL cryovial, immediately frozen on dry ice, and kept at − 80 °C until analysis. Fasting blood was evaluated for whole blood glucose, serum insulin, cortisol and cytokines, and plasma free fatty acids. Fecal samples were analyzed for microbiome community composition. At euthanasia, ~ five grams of subcutaneous adipose tissue (back fat) was collected, snap frozen and kept at − 80 °C for fatty acid composition and a section of the ileum was swabbed with a sterile swab placed in a 15-mL conical filled with 1 mL of ice cold phosphate buffered saline solution and snap frozen to be analyzed for microbiome community composition. Euthanasia was performed by the following protocol: intramuscular administration of 2 mL/20.4 kg body weight of telazol (Zoetis) -ketamine (Zetamine™, Vetone, Boise, ID, USA)-xylazine (Vetone), collection of 15 mL of blood from the cranial vena cava, and then intravenous administration of an overdose (1 mL/4.53 kg body weight) of pentobarbital (Fatal Plus, Vortech Pharmaceuticals Ltd., Dearborn, MI, USA).

### Metabolic parameter analysis

Serum insulin concentrations were measured with a porcine inulin radioimmunoassay (RIA) (Linco/Millipore Corporation, Billerica, MA, USA) which was validated by parallelism at the following volumes of pooled serum per tube: 100, 200, and 350 μL. For the insulin RIA, the intra-assay CV was 9.0% and the inter-assay CV was 8.3%. Fatty acids were extracted from diet samples and subcutaneous adipose tissue samples by a modified Folch method and identified by flame ionized gas chromatography [9]. Serum samples for the measurement of cortisol were extracted with diethyl ether and brought up in phosphate buffered saline solution [10]. To monitor extraction efficiency, 100 μL (1000 cpm/100 μL) of ^3^H-cortisol (Perkin Elmer, Waltham, MA, USA) was added to one mL of each serum sample [10]. Extraction efficiency for all samples was > 85%. Cortisol in each extraction was measured using a double antibody enzyme immunoassay (ELISA) (Cortisol ISWE mini-kit, Arbor Assays, Ann Arbor, MI, USA) which was validated by parallelism with 25, 50, 100 μL of pooled serum extraction. Raw cortisol concentration per sample from the ELISA was adjusted by sample extraction efficiency to calculate the actual cortisol concentration per sample. For the cortisol ELISA, the intra-assay CV was 8.0% and the inter-assay CV was 3.1%.

### Cytokine analysis

Cytokine concentrations [interleukin 1-beta (IL-1β), interleukin 10 (IL-10), interferon alpha (IFN-α), interferon gamma (IFN-γ), tumor necrosis Factor alpha (TNF-α), interleukin 4 (IL-4), and interleukin 8 (IL-8)] in serum were analyzed at each age using the Swine Cytokine Magnetic 7-Plex Panel assay (Novex®, Life Technologies Ltd., UK). Analysis of the assay was performed on a Luminex® 100/200™ in the Research Services Core at Southern Illinois University School of Medicine. For the IL-1β wells, the intra-assay CV was 6.6% and the inter-assay CV was 6.1%. For the IL-10 wells, the intra-assay CV was 15.4% and the inter-assay CV was 8.5%. For the IL-4 wells, the intra-assay CV was 13.2% and the inter-assay CV was 4.3%. For the IL-8 wells, the intra-assay CV was 3.4% and the inter-assay CV was 13.4%.

### Microbial assessment

Total genomic DNA was extracted from the fecal samples of all animals using the DNeasy PowerSoil Isolation kit from Qiagen (Germantown, MD, USA) according to the manufacturer’s instructions. Following DNA extraction, the stock concentration was quantified using a Qubit fluorometer (Invitrogen, Thermofisher Scientific, Carlsbad, CA, USA). To characterize the microbiome, we used Next Generation Sequencing (NGS) via an Illumina MiSeq system. Bacterial sequencing of the hypervariable V4 region of the 16S rRNA gene was performed with a two-step PCR approach to barcode tag templates with frame shifting nucleotide primers [11]. In the first PCR step, custom designed forward and reverse primer mixtures were used to maximize phylogenetic coverage of bacteria. In the second PCR step, barcodes were added to the templates for sequencing. Approximately 4,000,000 total sequence reads with 12,000 reads/sample were obtained, including both dominant and poorly-represented taxa of the gastrointestinal microbiome. Data were quality filtered and processed using a combined QIIME [12]/USEARCH [13] pipeline. Operational taxonomical units (OTU) were clustered at 97% sequence similarity and classified using BLAST and RDP reference databases for 16S bacteria. Differences in community composition and diversity were assessed across sampling times and between treatment groups.

### Statistical analysis

All metabolic, feed intake, and growth parameters were assessed for normality with PROC GLM (SAS, Cary, NC, USA). Non-normal variables were transformed with log 10 transformation for analysis by repeated measures ANOVA with pig id as random, age as the repeated variable, and diet as the fixed effect in the model (SAS). The co-variance structure applied in the model was compound symmetric. Parameter values reported in the results are back-transformed least squared means (LSM) and standard error of the means (SEM). The level of significance was set at *P* ≤ 0.05. A trend in significance was considered as 0.05<*P*< 0.10.

Cytokine concentrations at each timepoint for each animal were calculated as described herein. All samples were normalized to the background fluorescent intensity (no serum control; **B**) from the unknown sample fluorescent intensity (**US**), which was calculated from the standard curves provided in the assay kit. These values were then used to calculate the average for each animal (at each timepoint) across duplicate assays ((**US**1 – **B** + **US**2 – **B**)/2) = **X**. The averaged value for each sample (**X**) was then from used to calculate the average value for each treatment group (X_1_ + X_2_ + X_3_ … /*n*) and plotted on the representative graphs. Statistical differences between treatment groups was calculated by Mann-Whitney non-parametric analyses. The level of significance was set at *P* ≤ 0.05.

Microbial sequences were rarefied to a depth of 100 counts/ sample to ensure evenness of sampling. Clustering analysis was performed on rarified sequencing data through principal component analysis to determine beta diversity, or differences between the categorical variables: time (i.e. age), diet treatment, and sample type (ileal versus fecal). Significant differences between OTU counts for treatment groups were then determined through group differences Qiime script using the Kruskal Wallis (non-parametric) statistical testing with the level of significance set at *P* ≤ 0.05. Data shown for the microbial groups is the mean count per treatment group across all times (i.e. ages).

## Results

### Dietary composition

Dietary phases were formulated according to the nutritional demands at each stage of development (Additional file 1: Table S1), standardized for ileal digestibility (Additional file 1: Table S2) and had equal percentages of calcium and phosphorus (Additional file 1: Table S3) as recommended by swine nutritional guidelines. As substitution of soybean oil for coconut oil would alter the dietary fatty acid composition, fatty acids were measured in the diets. We found that the coconut oil diet (COC) had greater percentages of medium chain saturated fatty acids (caprylic, capric, lauric, and myristic acids) (Additional file 1: Table S4). However, this diet had decreased percentages of long chain saturated, monounsaturated and polyunsaturated fatty acids compared to dietary treatments with 2% soybean oil (ABX and NABX) (Additional file 1: Table S4).

### Growth performance

Overall the growth performance parameters between all diet treatment groups were similar. Furthermore, all significant differences in these parameters occurred within the first several weeks of the study. The ADFI was measured as the kilograms (kg) of feed ingested by each pig per day. At d31, as pigs transitioned to phase 2 diet, pigs receiving ABX had significantly greater feed intake than pigs receiving COC (Table 1). For ADFI there was a significant effect of diet treatment (Txt; *P*= 0.03) and age (Age; *P* <  0.001) in the model but not of Txt × Age (*P*= 0.65). As feed intake may not correlate with pig weight gain, the ADG of pigs was calculated as kgs of weight gain per pig per day. For ADG there was no significant effect of Txt (*P* = 0.15) nor of Txt × Age *P* = 0.99), but there was a significant effect of Age (*P* <  0.001) in the model. ADFI increases with age and the body frame of the pig as it grows, it follows that ADG would also increase with increasing age.

**Table 1:** Growth parameters for pigs by age and diet treatment^ab^

Age, d	ABX				COC				NABX			
	ADFI	ADG	FCR	WTG	ADFI	ADG	FCR	WTG	ADFI	ADG	FCR	WTG
31	0.27 ± 0.02*	0.13 ± 0.04	2.42 ± 0.52^#^	0.90 ± 0.30	0.22 ± 0.02*	0.12 ± 0.04	2.44 ± 0.54^#^	0.81 ± 0.30	0.24 ± 0.02	0.11 ± 0.04	3.36 ± 0.56^#^	0.79 ± 0.31
38	0.45 ± 0.02	0.32 ± 0.04	1.56 ± 0.52^#^	2.26 ± 0.30^&^	0.43 ± 0.02	0.27 ± 0.04	2.34 ± 0.53^*#*^	1.86 ± 0.30^&^	0.47 ± 0.02	0.29 ± 0.04	2.12 ± 0.5	2.06 ± 0.31
45	0.83 ± 0.02	0.52 ± 0.04	1.67 ± 0.52	3.61 ± 0.30	0.80 ± 0.02	0.44 ± 0.04	1.94 ± 0.53	3.07 ± 0.30	0.78 ± 0.02	0.47 ± 0.04	1.95 ± 0.54	3.30 ± 0.31
52	0.73 ± 0.02	0.64 ± 0.04	1.03 ± 0.52	4.51 ± 0.30	0.70 ± 0.02	0.64 ± 0.04	1.02 ± 0.53	4.46 ± 0.30	0.75 ± 0.02	0.62 ± 0.04	1.10 ± 0.54	4.31 ± 0.31
59	0.65 ± 0.02	0.80 ± 0.04	0.84 ± 0.52	5.58 ± 0.30	0.65 ± 0.02	0.75 ± 0.04	0.88 ± 0.53	5.23 ± 0.30	0.66 ± 0.02	0.74 ± 0.04	0.92 ± 0.54	5.15 ± 0.31
66	0.67 ± 0.02	0.60 ± 0.04	0.74 ± 0.52	4.19 ± 0.30	0.64 ± 0.02	0.59 ± 0.04	0.74 ± 0.53	4.11 ± 0.30	0.64 ± 0.02	0.51 ± 0.04	0.77 ± 0.54	3.54 ± 0.31

^a^ABX: 2% soybean oil with antibiotics; COC: 2% coconut oil without antibiotics; NABX: 2% soybean oil without antibiotics

^b^Animal and feed weights used for calculations are in kilograms

*ADFI* Average daily food intake (kg feed), *ADG* Average daily gain (kg weight), *FCR* Feed conversion ratio, *WTG* Weight gain (kg)/pig/week

* Within ADFI (column) and age (row), values are significantly different (*P* ≤ 0.05)

^#^ Within FCR (column) and age (row), values are significantly different (*P* ≤ 0.05)

^&^ Within WTG (column) and age (row), values are significantly different (*P* ≤ 0.05)

The feed conversion ratio (FCR) was calculated by measurement of kg of feed consumed per kg of body weight gained per pig. The smaller the FCR, the less food consumed (kg) by a pig per gain in body weight (kg). For FCR there was no significant effect of Txt (*P* = 0.34) nor of Txt × Age *(P* = 0.72), but there was a significant effect of Age (*P* <  0.001) in the model. At d31 (7 d on diet treatment), ABX and COC animals had significantly lower FCR than those pigs that were in the NABX treatment group. However, on d38 (14 d on diet treatment), COC pigs had significantly greater FCR than the ABX pigs. For pig weight gain (kgs weight gained/pig/week; WTG) there was no significant effect of Txt (*P* = 0.15) nor of Txt × Age (*P* = 0.99), but there was a significant effect of Age (*P* <  0.001) in the model. At d38, ABX pigs had significantly greater WTG than COC pigs.

### Metabolic parameters

Fatty acid composition in the subcutaneous white adipose tissue (“back fat”) was conducted to determine actual percentages of fatty acids in white adipose tissue after ingestion of the various diet treatments. Similar to the fatty acid composition of the coconut oil containing diet, the back fat of pigs fed the coconut oil fat had greater percentages of medium chain saturated fatty acids (Table 2) and decreased percentages of long chain mono- and polyunsaturated fatty acids. The exception was that vaccenic acid, a long chain *trans-* fatty acid, was lower in the COC diet but was greater in the back fat of COC-fed pigs. The overall main effect of Txt was significant in the model for analysis of white adipose tissue fatty acids (*P* <  0.001).

**Table 2:** Percentage of fatty acid acids in subcutaneous adipose tissue by diet treatment^1^

Common name	Formula	ABX	COC	NABX	*P*-value
Lauric	12:0	0.05 ± 0.03^a^	1.37 ± 0.03^b^	0.03 ± 0.03^a^	< 0.001
Myristic	14:0	1.46 ± 0.09^a^	4.66 ± 0.09^b^	1.29 ± 0.03^a^	< 0.001
Myristoleic	14:1	0.02 ± 0.01^a^	0.10 ± 0.01^b^	0.005 ± 0.01^a^	< 0.001
Palmitic	16:0	22.51 ± 0.24^a^	25.92 ± 0.24^b^	22.53 ± 0.24^a^	< 0.001
Palmitoleic	16:1	2.54 ± 0.12^a^	3.68 ± 0.12^b^	2.43 ± 0.12^a^	< 0.001
Stearic	18:0	11.21 ± 0.30^a^	11.66 ± 0.30^a^	11.06 ± 0.30^a^	0.71
Oleic	18:1n-9	31.57 ± 0.47^a^	32.65 ± 0.47^a*^	31.45 ± 0.45^a*^	0.07
*cis*-Vaccenic	18:1n-7	2.40 ± 0.08^a^	2.86 ± 0.08^b^	2.25 ± 0.08^a^	< 0.001
Linoleic	18:2n-6	23.60 ± 0.43^a^	14.59 ± 0.42^b^	24.54 ± 0.41^a^	< 0.001
*α*-Linolenic	18:3n-3	2.26 ± 0.06^a^	0.96 ± 0.06^b^	2.20 ± 0.06^a^	< 0.001
Arachidic	20:0	0.23 ± 0.01^a^	0.20 ± 0.01^a^	0.20 ± 0.01^a^	0.54
Paulinic	20:1n-11	0.51 ± 0.03^a^	0.42 ± 0.03^a^	0.48 ± 0.03^a^	0.48
Eicosadienoic	20:2n-6	0.82 ± 0.03^a^	0.37 ± 0.03^b^	0.76 ± 0.03^a^	< 0.001
Arachidonic	20:4n-6	0.45 ± 0.01^a^	0.38 ± 0.01^b^	0.44 ± 0.01^a^	0.01
Mead	20:3n-6	0.24 ± 0.01^a^	0.11 ± 0.01^b^	0.23 ± 0.01^a^	< 0.001
Docosahexaenoic	22:6n-3	0.11 ± 0.01^a^	0.08 ± 0.01^b^	0.10 ± 0.01^a^	0.01

^1^ABX: 2% soybean oil with antibiotics; COC: 2% coconut oil without antibiotics; NABX: 2% soybean oil without antibiotics

^ab^ Within rows, values with common superscripts are not different (*P* > 0.05)

* Within rows, values with asterisks tend to be different (*P* ≤ 0.10)

**Fig. 1:**
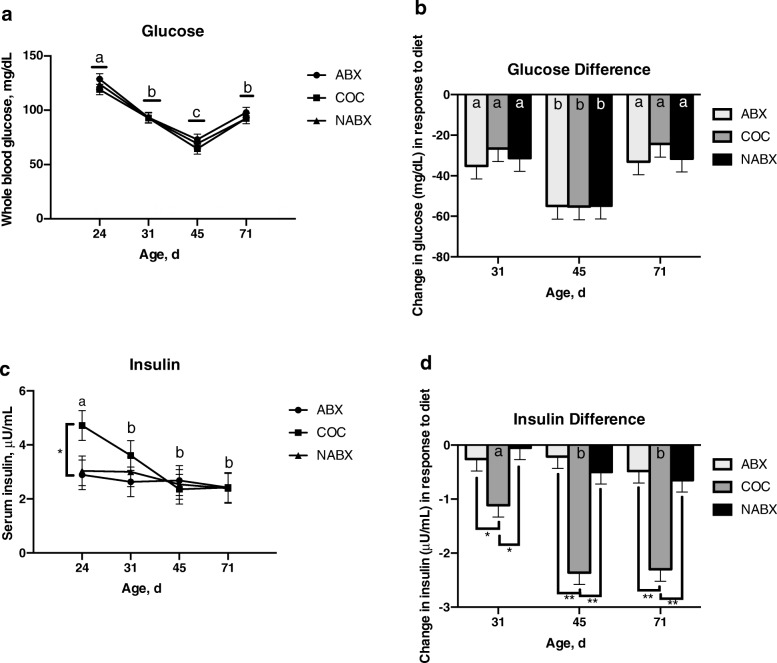
Effect of diet treatment on whole blood glucose and serum insulin. **a** Glucose levels in each diet treatment group (ABX, COC, and NABX) over time. **b** Change in glucose levels in response to diet over time. **c** Insulin levels in each diet treatment group (ABX, COC, and NABX) over time. **d** Change in insulin levels in response to diet over time. Values for the change in glucose or insulin were normalized to individual animal baseline (d24) measurements and then averaged across treatment groups for each age (d31, d45, d71). Within diet treatment but between age, points or bars with different lowercase letters have significant differences between ages within a diet treatment (*P* ≤ 0.05). Within age but between diet treatment, brackets with: * denotes significant differences between diet treatments at *P* ≤ 0.05; ** denotes significant differences between diet treatments at *P* ≤ 0.01

Irrespective of dietary treatment group, whole blood glucose concentrations decreased as pigs transitioned from weaning through phase 1 (d31) and 2 (d45) of the nursery period (Fig. 1a). Whole blood glucose concentrations increased during the grower stage of development (d71) but did not reach the same levels as post-weaning (d24). For whole blood glucose, there was a trend for the main effect of Txt (*P* = 0.10) and the main effect of Age was significant (*P* <  0.001) in the model but the interaction of Txt × Age was not significant (*P* = 0.015). The average change in blood glucose in response to diet was measured by subtraction of the initial, baseline glucose (d24) from each consecutive timepoint (d31, d45 & d71) for each pigs and then averaging across each treatment group (i.e., d31-d24 = change in glucose at d31). The change in whole blood glucose concentrations by age was greatest at the transition from phase 2 to phase 3 diet (Fig. 1b). However, the change in whole blood glucose concentrations was not different between diet treatments within age. For the difference in whole blood glucose concentrations, there was no significant effect of Txt (*P* = 0.55) or Txt × Age (*P* = 0.94) in the model but Age (*P* <  0.001) had a significant effect.

Serum insulin concentrations and the change in serum insulin concentrations in diet treatment groups was measured throughout the study. Pigs randomly assigned to the COC diet treatment had greater baseline concentrations of serum insulin than pigs in the ABX or NABX diet treatments (Fig. 1c). However, serum insulin concentrations in the COC treatment dropped as pigs started phase 1 diet and continued through phase 2 and 3 diets. For serum insulin, there was no significant effect of Txt (*P* = 0.22) or Txt × Age (*P* = 0.29) in the model but Age had a significant effect (*P* = 0.01). Pigs in the COC treatment had significantly reduced concentrations of insulin at each age reflected by the greater decrease in insulin at d31, d45 and d71 in COC pigs than the ABX and NABX pigs (Fig. 1d). Moreover, for the difference in serum insulin over time, all parameters in the model were significant (Txt: *P* <  0.001; Age *P* <  0.001; Txt × Age *P* = 0.01). These data indicate that consumption of coconut oil, as opposed to soybean oil, lowers serum insulin concentrations.

**Fig. 2:**
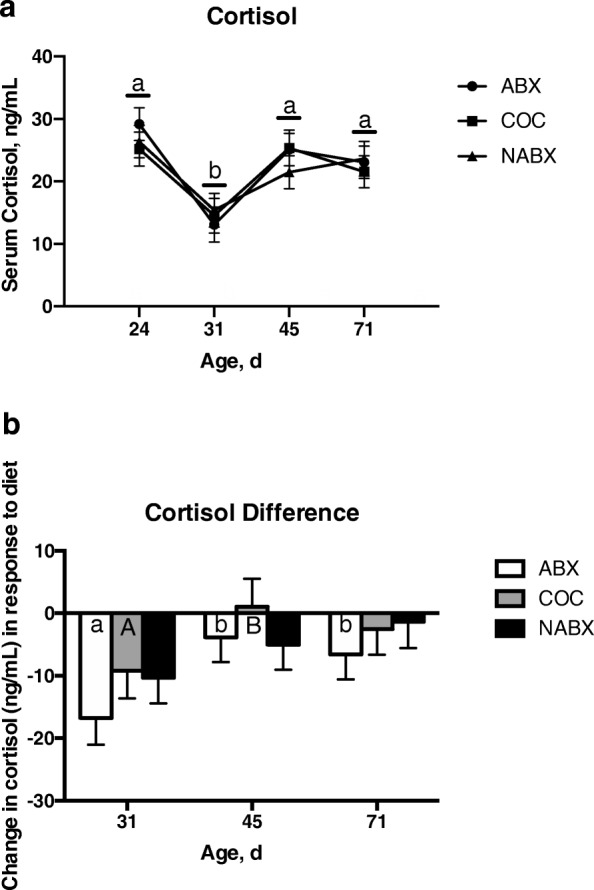
Effect of diet treatment on serum cortisol. **a** Cortisol levels in each diet treatment group ABX, COC, and NABX) over time. **b** Change in cortisol levels in response to diet over time. Values for the change in glucose or insulin were normalized to individual animal baseline (d24) measurements and then averaged across treatment groups for each age (d31, d45, d71). Within diet treatment but between age, points or bars with different lowercase letters denote significant differences between ages (*P* < 0.05) and bars with different uppercase letters denote a trend toward significant differences between ages (0.05 < *P* < 0.10)

To determine if endogenous function of the adrenal cortex differed between pigs on different dietary treatments, serum cortisol concentrations were measured throughout the study. Serum cortisol decreased for all dietary treatments at the transition to phase 2 diet (d31) (Fig. 1a). However, for a given age, there was no difference in serum cortisol between dietary treatments. For serum cortisol there was no significant effect of Txt (*P* = 0.81) nor of Txt × Age (*P* = 0.72), but there was a significant effect of Age (*P* <  0.001) in the model. The change in serum cortisol concentrations by age was greatest at the transition from phase 2 to phase 3 diet for ABX animals (Fig. 2b). For the difference in serum cortisol there was no significant effect of Txt (*P* = 0.27) nor of Txt × Age *(P* = 0.86), but there was a significant effect of Age (*P* = 0.01) in the model.

### Serum cytokines

**Fig. 3:**
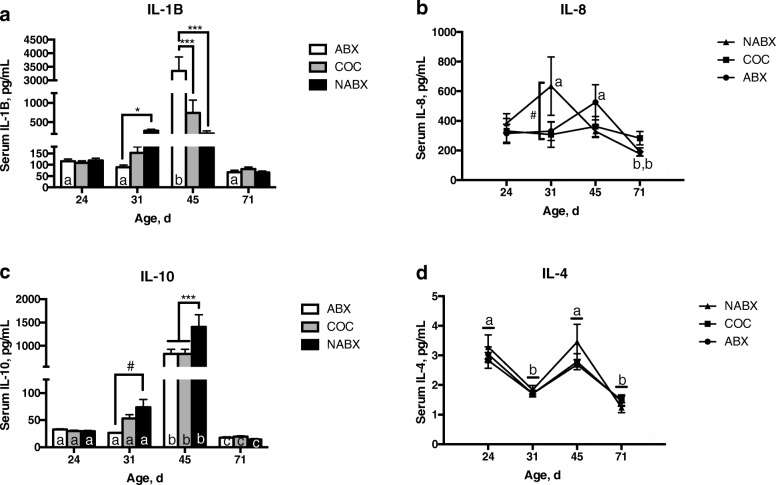
Effect of diet treatment on serum cytokines. **a** Interleukin 1β **b** Interleukin 8 **c** Interleukin 10 and **d** Interleukin-4 levels were measured. Within diet treatment but between age, points or bars with different lowercase letters denote significant differences between ages but within diet treatment (*P* ≤ 0.05). Within age but between diet treatment, brackets with: # denotes a trend for a significant difference between diet treatment (0.05 < *P* ≤ 0.10); * denotes significant differences between diet treatments at *P* ≤ 0.05; *** denotes significant differences between diet treatments at *P* ≤ 0.001

To evaluate the systemic immunological status of pigs on different diets, serum cytokines were assessed over time. Serum TNF-α, IFN-α and IFN-γ were below the detection limits of the cytokine array used. IL-1β and IL-8 are pro-inflammatory cytokines whereas IL-10 and IL-4 are anti-inflammatory cytokines. Serum IL-1β concentrations increased significantly at d45 of age in the ABX-fed pigs (Fig. 3a). At d31 of age, pigs fed NABX had significantly greater serum IL-1β concentrations than pigs fed ABX. By contrast, at d45 of age, pigs fed ABX had significantly greater serum IL-1β concentrations than either COC or NABX fed pigs. By d71 serum IL-1β concentrations were similar to the baseline levels at d24 across all diet treatments. For IL-1β, all parameters in the model were significant (Txt: *P* <  0.001; Age *P* < 0.001; Txt × Age *P* < 0.001).

Serum IL-8 concentrations were significantly greater in NABX fed pigs than either COC or ABX fed pigs at d31 of age (Fig. 3b). For NABX pigs, IL-8 concentrations were greater at d31 than d71. For ABX pigs, IL-8 concentrations were greater at d45 than d71. For COC pigs the concentrations of both pro-inflammatory cytokines were relatively similar regardless of pig age. For IL-8, Age was a significant parameter in the model (*P* = 0.01) and there was a trend for the interaction of Txt × Age (*P* = 0.06) but Txt was not significant (*P* = 0.60).

Across all diet treatments, the concentrations of IL-10 were greatest at d45 and lowest at d71 (Fig. 3c). NABX pigs had a trend for increased IL-10 as compared with ABX pigs at d31. NABX pigs had significantly greater IL-10 at d45 as compared with either ABX or COC pigs. For IL-10, all parameters in the model were significant (Txt: *P* = 0.02; Age *P* < 0.001; Txt × Age *P* = 0.001). Irrespective of diet treatment, IL-4 was greater on d24 and d45 and lower on d31 and d71 (Fig. 3d). There were no significant differences in serum IL-4 between treatment group with a given age. For IL-4, Age was a significant parameter in the model (*P* < 0.001) but Txt (*P* = 0.34) and its interaction Txt × Age (*P* = 0.62) were not significant.

### Microbial analysis

**Fig. 4:**
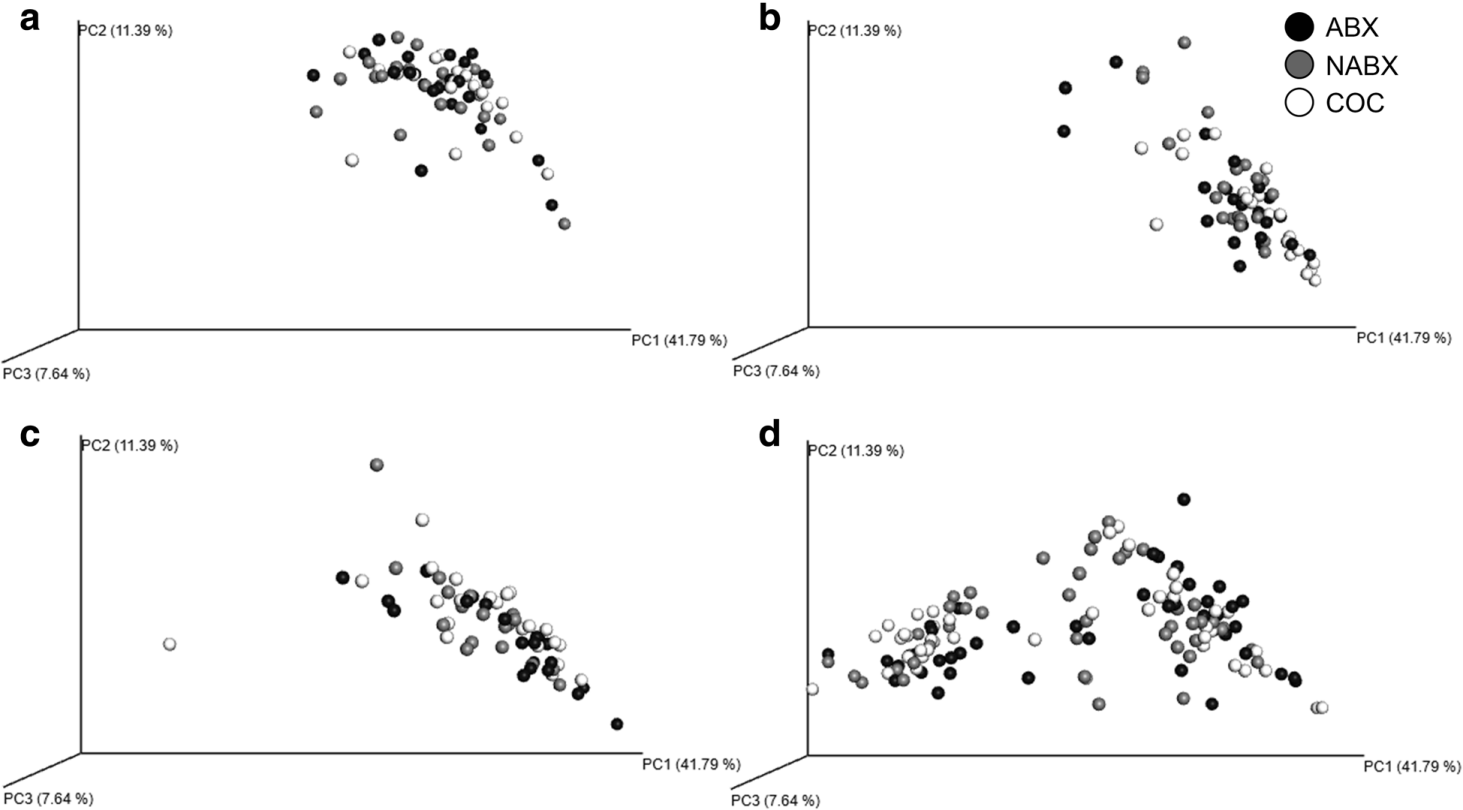
Principal component analysis (non-dimensional scaling) on microbial sequences from NGS. 3D clustering patterns of individual animals within each diet treatment by age. Black (ABX), grey (NABX) and white (COC) denote diet treatment. **a** d24 of age. **b** d31 of age. **c** d45 of age. **d** d71 of age. Spatial proximity of each dot indicates the similarity of one individual’s microbial communities to another individual’s microbial communities. The closer dots are the more similar samples are and the farther dots are the less similar samples are

**Fig. 5:**
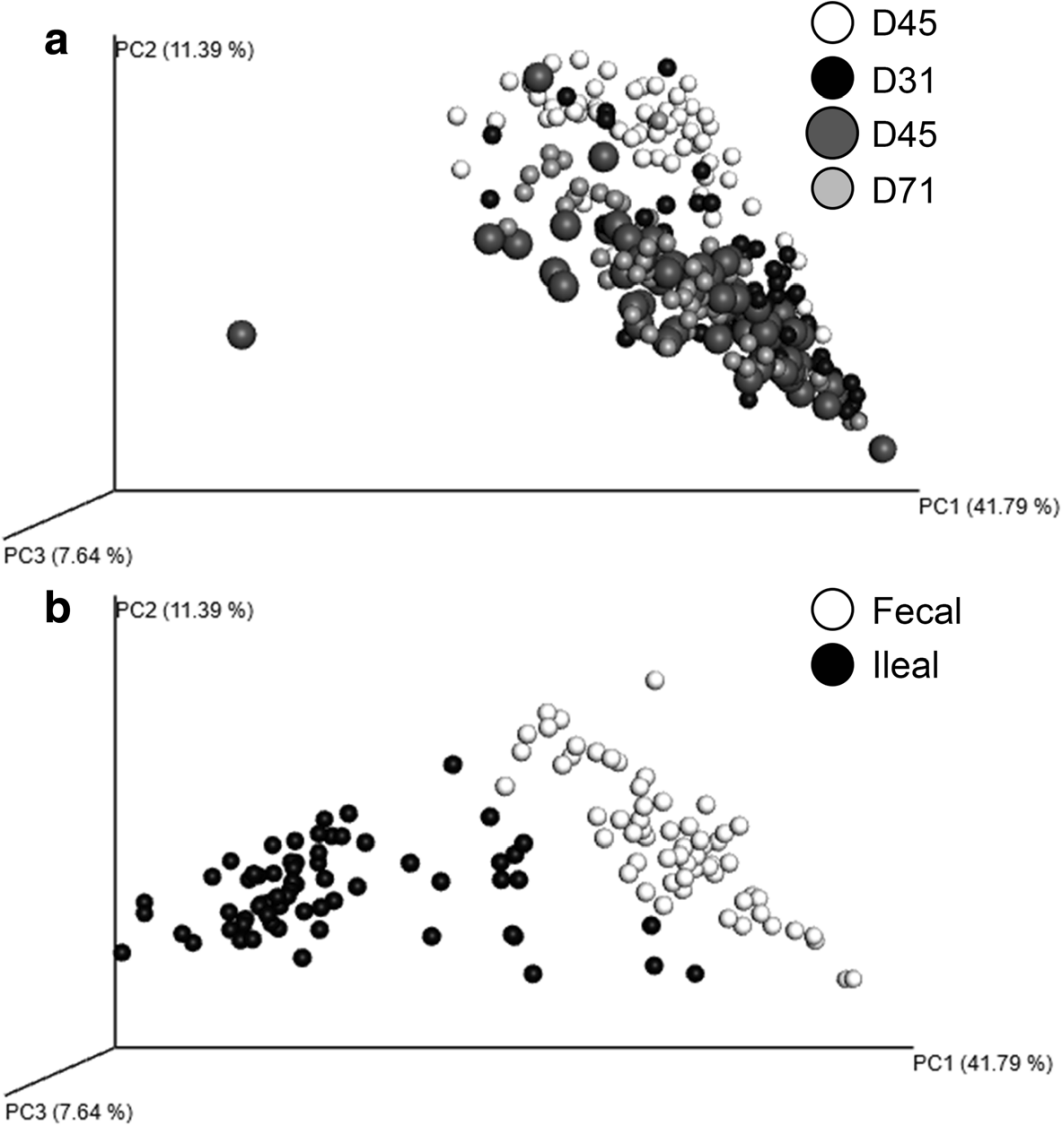
Effect of diet treatment on fecal and ileal microbial community dynamics. Principal component analysis of fecal and ileal samples was performed. **a** Combined analysis of samples collected by age. **b** Analysis of fecal and ileal sample microbial dynamics at d71 of age

As diet and nutrition affect the gut normal flora and local immunity, the fecal bacterial microbial composition was assessed for each diet at each age. Through beta diversity clustering, which analyzes similarity of microbial communities between categorical variables, we found no effect of diet treatment on microbial composition (Fig. 4a-d; *P* = 0.80). All timepoints on diet trial (d31-mid-phase 1 diet, d45-end-phase 1 diet, d71-end-phase 2 diet) had similar microbial clustering patterns (Fig. 5a; *P* = 0.68). However, the microbial clustering pattern at d24 of age or weaning was distinctly separate from that pattern seen throughout the diet trial. At the conclusion of the study on d71, ileal samples had separate and distinct microbial clustering patterns as compared with fecal samples (Fig. 5b; *P* = 0.04).

Although discrete clustering patterns were not detected by diet treatment, COC fed pigs had 117 differentially expressed OTU’s compared to ABX and NABX fed pigs. Using a threshold cutoff of two fold, we then narrowed the list of differentially expressed OTU’s to 37. Of these 37 OTU’s, 22 were expressed higher in COC-fed pigs and 15 were expressed higher in ABX or NABX fed pigs. Firmicutes and Bacteroidetes were the two major phyla dysregulated by diet treatment (Table 3).

**Table 3:** Differentially expressed bacterial OTU copy number in fecal samples by diet treatment^1^

ABX	COC	NABX	*P*-value	Bacterial taxonomy
389^a^	887^b^	404^a^	0.04	Bacteroidetes; Bacteroidia; Prevotellaceae; *Prevotella*
153^a^	293^b^	227^ab^	0.02	Bacteroidetes; Bacteroidia; Bacteroidales; Bacteroidales S24–7
46^a^	216^b^	89^a^	0.01	Firmicutes; Clostridia; Clostridiales; Lachnospiraceae; *Ruminococcus torques* group
15^a^	201^b^	125^b^	0.01	Firmicutes; Clostridia; Clostridiales; Peptostreptococcaceae; *Peptoclostriudium*
1^a^	23^b^	12^ab^	0.01	Firmicutes; Erysipelotrichia; Erysipelotrichales; Erysipelotrichaceae; *Turicibacter*
1^a^	18^ab^	53^b^	0.02	Bacteroidetes; Bacteroidia; Bacteroidales; Porphyromonadaceae; *Parabacteroides*
2^a^	12^b^	4^ab^	0.03	Firmicutes; Clostridia; Clostridiales; Lachnospiraceae
3^a^	0.81^b^	0.8^b^	0.03	Bacteroidetes; Bacterodidia; Bacteroidales; Prevotellaceae; *Prevotellaceae UCG-100*
3.7^a^	0.53^b^	0.92^b^	0.03	Firmicutes; Clostridia; Clostridailaes; Lachnospiraceae; *Coprococcus*

^1^ABX: 2% soybean oil with antibiotics; COC: 2% coconut oil without antibiotics; NABX: 2% soybean oil without antibiotics; OTU: average over all ages is shown for each diet treatment

^ab^ Within rows, values with common superscripts are not different (*P* > 0.05)

## Discussion

Pressure both internally from U.S. citizens and the American government and externally from international importers has forced the swine industry to assess its use of growth-promoting antibiotics over the past 10–15 years. This study investigated the potential application of dietary coconut oil as a substitute for antibiotic feed additives. In brief, pigs given dietary coconut oil had similar growth rates and fecal microbial dynamics compared to both those given antibiotic feed additives and those fed a diet without antibiotic additives. Therefore, this work indicates that antibiotics are not necessary for the maintenance of general health, growth, and gut microbial dynamics in pigs.

The majority of differences within growth parameters between diet treatment groups occurred within the first 7–14 d on the diet trial, corresponding with the critical post-weaning period. The FCR was greater for the NABX pigs in the first week post-weaning as compared to either ABX or COC pigs. Although we did not note severe diarrhea in any treatment group at any point during the study, the NABX pigs did have more watery stools the first 2 weeks post-weaning and on diet trial. It is possible that this group was not as efficient at growth around the critical weaning time period. In fact, the weight of pigs at weaning and the diet onto which they are creep-fed have long-term effects on the growth performance of pigs [14]. On the other hand, by the age of 38 d or 14 d on diet trial, COC pigs had a higher FCR and a lower weight gain than the ABX group. This could be due to an effect of the COC itself or it could be indirect due to a dislike for the flavor of coconut oil leading to decreased feed consumption. Irrespective of these initial differences in growth parameters between the groups, over the course of the study the growth parameters became very similar between the treatment groups. This finding indicates that not only is dietary coconut oil not necessarily beneficial to growth in pigs but that antibiotic supplementation does not appear to be necessary either. In fact several other studies have demonstrated that pigs receive minimal [15, 16] or no benefit [17] from dietary antibiotic supplementation as long as the animals are housed in an “all-in, all-out” system with good biosecurity.

All diet treatments had a decrease in fasting glucose at 45 d of age; however, only the COC-fed pigs had a decrease in fasting serum insulin levels at 45 and 71 d of age. During this same time period (d45), ADFI increased dramatically for all treatment groups. It is possible that increased efficiency of glucose uptake into cells around this time period resulted in decreased fasting glucose and increased food intake. Coconut oil has been shown to improve insulin sensitivity in diet-induced insulin resistant rats [18] and glycemic control in obese pigs [19]. Therefore, our study suggests that dietary coconut oil may also improve insulin sensitivity in lean animals. The coconut oil diet was enriched in medium chain fatty acids (MCFA) and had less polyunsaturated fatty acids (PUFA) compared to the ABX and NABX diets which contained soybean oil. Therefore, it is not surprising that COC-fed pigs had an increased percentage of MCFA and saturated long chain fatty acids (LCFA) in their back fat compared to soybean oil-fed pigs. Interestingly, the back fat of COC-fed pigs had decreases in both omega 3 and omega 6 PUFAs compared to the back fat of either the ABX- or NABX-fed pigs. However, the back fat of COC-fed pigs had increased amounts of vaccenic acid as compared with ABX and NABX-fed pigs. Vaccenic acid, a *cis-*isomer of oleic acid, is naturally found in dairy products and meat from ruminant animals [20]. Vaccenic acid is a precursor of conjugated linoleic acid (CLA) and may contain anti-atherosclerotic properties and improve body composition [21] and lipid profile [22].

A healthy gut microbiome is paramount to adequate systemic immune health. Several studies have demonstrated that microbial supplementation bolsters gut health and immune function in the presence of infection [23–28]. Modulation of gut immunity during the critical windows of weaning and creep feeding has the most influence on innate immunity and may maximize pig growth performance [29]. While the interrelationship between microbial dynamics and gastrointestinal immune regulation has been investigated previously in neonatal pigs [30, 31], our study focused on dietary intervention in post-weaning pigs not influenced by the maternal microbiome. Although an overall effect of diet treatment on fecal microbiome was not found, the fecal microbiome on d24 was distinctly different from all other age timepoints. The sow microbiome has a significant influence on the suckling piglet fecal microbiome [32] which likely resulted in the different clustering of the microbiome for d24 (day of weaning) as opposed to d31, d45 and d71. However, by 7 d post-weaning or d31 of age, the fecal microbiome had become substantially different from the fecal microbiome on the day of weaning or d24 of age. This finding corresponds with the literature which has found that the maturation of the piglet fecal microbiota stabilizes by 10 days of age [33].

There was no significant change in microbial dynamics in response to a diet containing coconut oil, but rather a shift in the microbiome of COC-fed pigs as compared with ABX- and NABX-fed pigs. Similar to studies of other potential dietary antibiotic substitutes, we found a higher ratio of Bacteroidetes:Firmicutes and specifically higher *Prevotella* and Lachnospiraceae [34, 35]. Interestingly, this study also found an increase in Ruminococcaceae species which are important for caproic acid utilization and production [36]. Taken together, these data indicate that while microbial community dynamics were not altered by diet treatment, alteration of distinct microbial taxa occurred in response to coconut oil as opposed to soybean oil. Unsurprisingly, there was a different clustering of the ileal (small intestine) as opposed to fecal (large intestine) microbiome which corresponds to what has been found previously for these types of samples [37].

Although a study of the effect of dietary coconut oil on systemic inflammation has not been conducted, MCT fed to obese male mice has been shown to ameliorate insulin resistance and systemic inflammation approximated by IL-6 serum levels [38]. COC-fed pigs had similar concentrations of serum IL-8, a pro-inflammatory cytokine made by macrophages which is chemotactic for neutrophils throughout the study. However, this cytokine peaked for NABX-fed pigs on d31 and for ABX-fed pigs on d45. Similarly, IL-1*β*, another pro-inflammatory cytokine, peaked in the serum on d31 in NABX-fed pigs and on d45 there was a massive increase in the ABX-fed pigs. This dramatic change in serum IL-1β concentrations is unlikely to be related to cortisol, because cortisol concentrations were consistent among diet treatments at d45. As mentioned above, severe diarrhea was not noted in any treatment group during the study nor were any pigs severely ill. The large increase in serum IL-1*β*, in the ABX-fed group is possibly indicative of a subclinical infection in that group. Therefore, COC-fed pigs did not have elevated pro-inflammatory cytokine levels compared with pigs fed either the ABX or NABX diets. These results demonstrate that the ABX pigs had their greatest concentrations of circulating pro-inflammatory cytokines at d45, whereas for NABX pigs their greatest concentrations of circulating pro-inflammatory cytokines were at d31. Interestingly, the NABX-fed pigs had increases in the anti-inflammatory cytokine, IL-10 on d31 and d45 of the study. By contrast serum levels of the anti-inflammatory cytokine, IL-4, were quite low in all diet treatments throughout the study and minimally changed. As these cytokines are important to T cell differentiation and function and the adaptive immune response, the serum IL-10 and IL-4 data indicate that dietary coconut oil did not impede anti-inflammatory function.

Interestingly, although there was not a difference in serum cortisol levels between diet treatments, there was a dip in serum cortisol for all diet treatments at 31 d of age or 7 d post-weaning. Although salivary cortisol levels in growing pigs have been shown to decrease steadily overtime post-birth [39], a decrease at 31 d of age followed by a rebound thereafter has not been previously demonstrated. This decrease in serum cortisol although statistically significant is minimally significant from a physiologic perspective as it not correlated with changes in either growth or immune function parameters.

## Conclusions

Overall these data demonstrate that antibiotic feed supplementation does not improve overall immunity and general health nor does it bolster growth of growing pigs as compared with either dietary coconut oil or soybean oil. As dietary coconut oil does not improve growth performance and would be an added cost to the producer, we do not recommend it as a replacement for growth-promoting antibiotics. Therefore, as long as good biosecurity is maintained, subclinical dosages of antibiotics in feed rations are not necessary to optimize health and growth in young pigs nor does dietary coconut oil improve these parameters.

## Supplementary information

**Additional file 1: Table S1.** Pig diet composition by diet phase. **Table S2.** Standardized ileal digestibility. **Table S3.** Calcium and phosphorus percentages. **Table S4.** Percentage of fatty acid acids in each diet treatment.

## Data Availability

The datasets used and/or analyzed during the current study are available from the corresponding author on reasonable request.

## Abbreviations

^3^H: Tritiated
ABX: Antibiotics treatment group
ADFI: Average daily feed intake
ADG: Average daily gain
B: Background fluorescent intensity
CLA: Conjugated linoleic acid
COC: Coconut oil treatment group
d24: Day 24 of age
d31: Day 31 of age
d45: Day 45 of age
d71: Day 71 of age
DNA: Deoxynucleic acid
EDTA: Ethylenediaminetetraacetic acid
ELISA: Enzyme-linked immunosorbent assay
FA: Fatty acids
FCR: Feed conversion ratio
IACUC: Institutional Animal Care and Use Committee
IFN-α: Interferon alpha
IFN-γ: Interferon gamma
IL-10: Interleukin 10
IL-1β: Interleukin 1-beta
IL-4: Interleukin 4
IL-8: Interleukin 8
LSM: Least squares means
MCFA: Medium chain fatty acids
MCT: Medium chain triglycerides
NABX: No antibiotics treatment group
NF-κB: Nuclear factor kappa-light-chain-enhancer of activated B cells
NGS: Next Generation Sequencing
OTU: Operational taxanomic unit
PCR: Polymerase chain reaction
PUFA: Polyunsaturated fatty acids
RIA: Radioimmunoassay
rRNA: Ribosomal ribonucleic acid
SEM: Standard error of the mean
TNF-α: Tumor necrosis Factor alpha
US: Unknown sample fluorescent intensity
W/W: Weight/weight
WTG: Animal weight gain
X: Averaged value for each sample

## References

[1] Dritz SS, Tokach MD, Goodband RD, Nelssen JL (2002). Effects of administration of antimicrobials in feed on growth rate and feed efficiency of pigs in multisite production systems. J Am Vet Med Assoc.

[2] Smith TC, Gebreyes WA, Abley MJ, Harper AL, Forshey BM, Male MJ (2013). Methicillin-resistant Staphylococcus aureus in pigs and farm workers on conventional and antibiotic-free swine farms in the USA. PLoS One.

[3] Services, U.S.D.o.H.a.H. Guidance for Industry #213: New Animal Drugs and New Animal Drug Combination Products Administered in or on Medicated Feed or Drinking Water of Food-Producing Animals: Recommendations for Drug Sponsors for Voluntarily Aligning Product Use Conditions with GFI #209, F.a.D. Administration, Editor. 2013: Center for Veterinary Medicine.

[4] Wanten GJ, Calder PC (2007). Immune modulation by parenteral lipid emulsions. Am J Clin Nutr.

[5] Hill TM, Vandehaar MJ, Sordillo LM, Catherman DR, Bateman HG, Schlotterbeck RL (2011). Fatty acid intake alters growth and immunity in milk-fed calves. J Dairy Sci.

[6] Bhatt RS, Soren NM, Tripathi MK, Karim SA (2011). Effects of different levels of coconut oil supplementation on performance, digestibility, rumen fermentation and carcass traits of Malpura lambs. Anim Feed Sci Technol.

[7] Jiao S, Maltecca C, Gray KA, Cassady JP (2014). Feed intake, average daily gain, feed efficiency, and real-time ultrasound traits in Duroc pigs: I. genetic parameter estimation and accuracy of genomic prediction. J Anim Sci.

[8] Newell-Fugate AE, Taibl JN, Clark SG, Alloosh M, Sturek M, Krisher RL (2014). Effects of diet-induced obesity on metabolic parameters and reproductive function in female Ossabaw Minipigs. Comparative Medicine.

[9] Smith SB, Go GW, Johnson BJ, Chung KY, Choi SH, Sawyer JE (2012). Adipogenic gene expression and fatty acid composition in subcutaneous adipose tissue depots of Angus steers between 9 and 16 months of age. J Anim Sci.

[10] Newell-Fugate AE, Taibl JN, Alloosh M, Sturek M, Bahr JM, Nowak RA (2015). Effects of obesity and metabolic syndrome on Steroidogenesis and Folliculogenesis in the female Ossabaw mini-pig. PLoS One.

[11] Lundberg DS, Yourstone S, Mieczkowski P, Jones CD, Dangl JL (2013). Practical innovations for high-throughput amplicon sequencing. Nat Methods.

[12] Caporaso JG, Kuczynski J, Stombaugh J, Bittinger K, Bushman FD, Costello EK (2010). QIIME allows analysis of high-throughput community sequencing data. Nat Methods.

[13] Edgar RC (2010). Search and clustering orders of magnitude faster than BLAST. Bioinformatics.

[14] Collins CL, Pluske JR, Morrison RS, McDonald TN, Smits RJ, Henman DJ (2017). Post-weaning and whole-of-life performance of pigs is determined by live weight at weaning and the complexity of the diet fed after weaning. Anim Nutr.

[15] Li J (2017). Current status and prospects for in-feed antibiotics in the different stages of pork production - a review. Asian Australas J Anim Sci.

[16] Diana A, Boyle LA, Leonard FC, Carroll C, Sheehan E, Murphy D (2019). Removing prophylactic antibiotics from pig feed: how does it affect their performance and health?. BMC Vet Res.

[17] Van Lunen TA (2003). Growth performance of pigs fed diets with and without tylosin phosphate supplementation and reared in a biosecure all-in all-out housing system. Can Vet J.

[18] Sun H, Jiang T, Wang S, He B, Zhang Y, Piao D (2013). The effect of LXRα, ChREBP and Elovl6 in liver and white adipose tissue on medium- and long-chain fatty acid diet-induced insulin resistance. Diabetes Res Clin Pract.

[19] Newell-Fugate AE, Lenz K, Skenandore C, Nowak RA, White BA, Braundmeier-Fleming A (2017). Effects of coconut oil on glycemia, inflammation, and urogenital microbial parameters in female Ossabaw mini-pigs. PLoS One.

[20] Field CJ, Blewett HH, Proctor S, Vine D (2009). Human health benefits of vaccenic acid. Appl Physiol Nutr Metab.

[21] Dilzer A, Park Y (2012). Implication of conjugated linoleic acid (CLA) in human health. Crit Rev Food Sci Nutr.

[22] Tricon S, Burdge GC, Jones EL, Russell JJ, El-Khazen S, Moretti E (2006). Effects of dairy products naturally enriched with cis-9,trans-11 conjugated linoleic acid on the blood lipid profile in healthy middle-aged men. Am J Clin Nutr.

[23] Ma T, Suzuki Y, Guan LL (2018). Dissect the mode of action of probiotics in affecting host-microbial interactions and immunity in food producing animals. Vet Immunol Immunopathol.

[24] Pu J, Chen D, Tian G, He J, Zheng P, Mao X (2018). Protective effects of benzoic acid, bacillus Coagulans, and oregano oil on intestinal injury caused by Enterotoxigenic Escherichia coli in weaned piglets. Biomed Res Int.

[25] Wang J, Zeng Y, Wang S, Liu H, Zhang D, Zhang W (2018). Swine-derived probiotic Lactobacillus plantarum inhibits growth and adhesion of Enterotoxigenic Escherichia coli and mediates host defense. Front Microbiol.

[26] Burdick Sanchez NC, Carroll JA, Broadway PR, Bass BE, Frank JW (2019). Supplementation of a Lactobacillus acidophilus fermentation product can attenuate the acute phase response following a lipopolysaccharide challenge in weaned pigs. Animal.

[27] Kim J, Kim J, Kim Y, Oh S, Song M, Choe JH (2018). Influences of quorum-quenching probiotic bacteria on the gut microbial community and immune function in weaning pigs. Anim Sci J.

[28] Pan L, Zhao PF, Ma XK, Shang QH, Xu YT, Long SF (2017). Probiotic supplementation protects weaned pigs against enterotoxigenic Escherichia coli K88 challenge and improves performance similar to antibiotics. J Anim Sci.

[29] Broom LJ, Kogut MH (2018). Gut immunity: its development and reasons and opportunities for modulation in monogastric production animals. Anim Health Res Rev.

[30] Maradiaga N, Aldridge B, Zeineldin M, Lowe J (2018). Gastrointestinal microbiota and mucosal immune gene expression in neonatal pigs reared in a cross-fostering model. Microb Pathog.

[31] Tsai T, Sales MA, Kim H, Erf GF, Vo N, Carbonero F (2018). Isolated rearing at lactation increases gut microbial diversity and post-weaning performance in pigs. Front Microbiol.

[32] Liu H, Zeng X, Zhang G, Hou C, Li N, Yu H (2019). Maternal milk and fecal microbes guide the spatiotemporal development of mucosa-associated microbiota and barrier function in the porcine neonatal gut. BMC Biol.

[33] Chen L, Xu Y, Chen X, Fang C, Zhao L, Chen F (2017). The maturing development of gut microbiota in commercial piglets during the weaning transition. Front Microbiol.

[34] Bin P, Tang Z, Liu S, Chen S, Xia Y, Liu J (2018). Intestinal microbiota mediates Enterotoxigenic Escherichia coli-induced diarrhea in piglets. BMC Vet Res.

[35] Zhu JJ, Gao MX, Song XJ, Zhao L, Li YW, Hao ZH (2018). Changes in bacterial diversity and composition in the faeces and colon of weaned piglets after feeding fermented soybean meal. J Med Microbiol.

[36] Wang H, Li X, Wang Y, Tao Y, Lu S, Zhu X (2018). Improvement of n-caproic acid production with Ruminococcaceae bacterium CPB6: selection of electron acceptors and carbon sources and optimization of the culture medium. Microb Cell Factories.

[37] Zhao W, Wang Y, Liu S, Huang J, Zhai Z, He C (2015). The dynamic distribution of porcine microbiota across different ages and gastrointestinal tract segments. PLoS One.

[38] Geng S, Zhu W, Xie C, Li X, Wu J, Liang Z (2016). Medium-chain triglyceride ameliorates insulin resistance and inflammation in high fat diet-induced obese mice. Eur J Nutr.

[39] Ruis MAW, Te Brake JHA, Engel B, Ekkel ED, Buist WG, Blokhuis HJ (1997). The circadian rhythm of salivary cortisol in growing pigs: effects of age, gender, and stress. Physiol Behav.

