# Supplementary material for: Dietary substitution of soybean oil with coconut oil in the absence of dietary antibiotics supports growth performance and immune function in nursery and grower pigs
Source: J Anim Sci Biotechnol. 2020 Mar 16;11:27. doi: 10.1186/s40104-020-0428-4 (PMC7075000; doi:10.1186/s40104-020-0428-4)
Supplement: Supplementary file 1 — Additional file 1: Table S1. Pig diet composition by diet phase. Table S2. Standardized ileal digestibility. Table S3. Calcium and phosphorus percentages. Table S4. Percentage of fatty acid acids in each diet treatment. [file 40104_2020_428_MOESM1_ESM.docx]

**Supplemental Materials**

| Table S1. Pig diet composition by diet phase | | | | | | |
| --- | --- | --- | --- | --- | --- | --- |
| Ingredients, % as fed |  | **Phase 1** |  | **Phase 2** |  | **Phase 3** |
| Corn |  | 43.45 |  | 50.65 |  | 66.15 |
| Soybean meal, Dehull, Sol Extr |  | 18.9 |  | 26.3 |  | 30.45 |
| Fish meal combined |  | 5 |  | 2.5 |  | - |
| Dried whey (& 2% lactose) |  | 25 |  | 15 |  | - |
| HP300 (Processed Soybean meal) |  | 5 |  | 2.5 |  | - |
| Calcium phosphate |  | 0.225 |  | 0.6 |  | 1.05 |
| Limestone, ground |  | 0.8 |  | 0.95 |  | 1.15 |
| Sodium chloride |  | 0.35 |  | 0.35 |  | 0.35 |
| *L*-Lys·HCl |  | 0.31 |  | 0.33 |  | 0.35 |
| *DL*-Met |  | 0.17 |  | 0.17 |  | 0.12 |
| *L*-Thr |  | 0.12 |  | 0.13 |  | 0.125 |
| VTM DMS |  | 0.25 |  | 0.25 |  | 0.25 |
| Quantaumblue 5g (phytase) |  | 0.015 |  | 0.01 |  | 0.015 |
| Zinc oxide |  | 0.4 |  | 0.25 |  | - |
| Total |  | 100 |  | 100 |  | 100 |

| **Table S2. Standardized ileal digestibility** |  |  |  |
| --- | --- | --- | --- |
| **Calculated composition SID amino acids, %** | **Phase 1** | **Phase 2** | **Phase 3** |
| Lysine | 1.38 | 1.35 | 1.2 |
| Isoleucine; lysine | 61 | 61 | 61 |
| Leucine; lysine | 103 | 112 | 127 |
| Methionine; lysine | 36 | 36 | 33 |
| Met & Cys; lysine | 57 | 57 | 56 |
| Threonine; lysine | 63 | 63 | 62 |
| Tryptophan; lysine | 17.4 | 17.8 | 1739 |
| Valine; lysine | 65 | 65 | 66 |
| Total lysine | 1.52 | 1.5 | 1.34 |

| **Table S3. Calcium and phosphorus percentages** |  |  |  |
| --- | --- | --- | --- |
| **Calculated composition of calcium and phosphorus percentages, %** | **Phase 1** | **Phase 2** | **Phase 3** |
| Calcium | 0.78 | 0.75 | 0.7 |
| Phosphorus | 0.65 | 0.64 | 0.61 |
| Available phosphorus | 0.49 | 0.46 | 0.42 |

| **Table S4. Percentage of fatty acid acids in each diet treatment^1^** | | | | |
| --- | --- | --- | --- | --- |
| **Common name** | **Formula** | **ABX** | **COC** | **NABX** |
| Caprylic | 8:0 | 0.00 ± 0.08^a^ | 0.22 ± 0.08^b^ | 0.00 ± 0.08^a^ |
| Capric | 10:0 | 0.00 ± 0.23^a^ | 0.40 ± 0.23^a^ | 0.00 ± 0.23^a^ |
| Lauric | 12:0 | 0.00 ± 0.47^a^ | 22.15 ± 0.47^b^ | 0.00 ± 0.47^a^ |
| Myristic | 14:0 | 0.58 ± 0.43^a^ | 9.65 ± 0.43^b^ | 0.59 ± 0.43^a^ |
| Myristoleic | 14:1n-5 | 0.025 ± 0.02^a^ | 0.035 ± 0.02^a^ | 0.035 ± 0.02^a^ |
| Palmitic | 16:0 | 13.06 ± 0.62^a^ | 12.94 ± 0.62^a^ | 13.05 ± 0.62^a^ |
| Palmitoleic | 16:1 | 13.06 ± 0.62^a^ | 12.94 ± 0.62^a^ | 13.05 ± 0.62^a^ |
| Stearic | 18:0 | 3.50 ± 0.18^a^ | 3.32 ± 0.18^a^ | 3.48 ± 0.18^a^ |
| Oleic | 18:1n-9 | 21.77 ± 0.25^a^ | 14.55 ± 0.25^b^ | 21.97 ± 0.25^a^ |
| *cis-*Vaccenic | 18:1n-7 | 1.18 ± 0.05^a^ | 0.62 ± 0.05^b^ | 1.17 ± 0.05^a^ |
| Linoleic | 18:2n-6 | 51.96 ± 1.87^a^ | 31.92 ± 1.87^b^ | 51.52 ± 1.87^a^ |
| γ-Linolenic | 18:3n-3 | 5.41 ± 0.32^a^ | 2.14 ± 0.32^b^ | 5.36 ± 0.32^a^ |
| Arachidic | 20:0 | 0.36 ± 0.005^a^ | 0.28 ± 0.005^b^ | 0.36 ± 0.005^a^ |
| Paulinic | 20:1n-11 | 0.24 ± 0.01^a^ | 0.19 ± 0.01^b^ | 0.24 ± 0.01^a^ |
| Eicosadienoic | 20:2n-6 | 0.022 ± 0.02^a^ | 0.00 ± 0.02^a^ | 0.025 ± 0.02^a^ |
| Arachidonic | 20:4n-6 | 0.078 ± 0.05^a^ | 0.075 ± 0.05^a^ | 0.07 ± 0.05^a^ |
| Behenic | 22:0 | 0.28 ± 0.01^a^ | 0.17 ± 0.01^b^ | 0.29 ± 0.01^a^ |
| Eicosapentaenoic | 20:5n-3 | 0.38 ± 0.24^a^ | 0.38 ± 0.24^a^ | 0.40 ± 0.24^a^ |
| Lignoceric | 24:0 | 0.22 ± 0.03^a^ | 0.16 ± 0.03^a^ | 0.23 ± 0.03^a^ |
| Nervonic | 24:1n-6 | 0.042 ± 0.04^a*^ | 0.067 ± 0.04^a^ | 0.16 ± 0.04^a*^ |
| Docosahexaenoic | 22:6n-3 | 0.34 ± 0.21^a^ | 0.36 ± 0.21^a^ | 0.49 ± 0.21^a^ |

^1^ ABX: 2% soybean oil with antibiotics; COC: 2% coconut oil without antibiotics; NABX: 2% soybean oil without antibiotics

^ab^ Within rows, values with common superscripts are not different (*P* > 0.05)
